# Retraction: Synthesis and characterization of a supported Pd complex on volcanic pumice laminates textured by cellulose for facilitating Suzuki–Miyaura cross-coupling reactions

**DOI:** 10.1039/d4ra90042a

**Published:** 2024-04-17

**Authors:** Siavash Salek Soltani, Reza Taheri-Ledari, S. Morteza F. Farnia, Ali Maleki, Alireza Foroumadi

**Affiliations:** a School of Chemistry, College of Science, University of Tehran Tehran Iran mfarnia@khayam.ut.ac.ir +98 2166495291; b Catalysts and Organic Synthesis Research Laboratory, Department of Chemistry, Iran University of Science and Technology Tehran 16846-13114 Iran maleki@iust.ac.ir +98-21-73021584 +98-21-77240540-50; c Drug Design and Development Research Center, The Institute of Pharmaceutical Sciences (TIPS), Tehran University of Medical Sciences Tehran Iran aforoumadi@yahoo.com +98 2166954708; d Department of Medicinal Chemistry, Faculty of Pharmacy, Tehran University of Medical Sciences Tehran Iran

## Abstract

Retraction of ‘Synthesis and characterization of a supported Pd complex on volcanic pumice laminates textured by cellulose for facilitating Suzuki–Miyaura cross-coupling reactions’ by Siavash Salek Soltani *et al.*, *RSC Adv.*, 2020, **10**, 23359–23371, https://doi.org/10.1039/D0RA04521G.

The Royal Society of Chemistry hereby wholly retracts this *RSC Advances* article due to concerns with the reliability of the data reported in Fig. 4b of the article and the NMR spectra in the electronic supplementary information (ESI).

Fig. 4b of this article is identical to Fig. 2b in ref. 1 and Fig. 4d of this article is identical to Fig. 3f in ref. 2.

An independent expert has reviewed both this article and ref. 1 and has raised concerns with the NMR data in the ESI. Several of the NMR spectra have strange features, for example, baselines with small gaps and a lack of noise in the baselines.

The authors have not been able to provide the original raw NMR data, so we are unable to verify the reliability of the published data.

Given the significance of the concerns about the validity of the data and the lack of raw data, the findings presented in this paper are not reliable.

The authors were informed about the retraction of the article but have not responded.

Signed: Laura Fisher, Executive Editor, *RSC Advances*

Date: 10th April 2024

## Supplementary Material
